# PhotoAgeClock: deep learning algorithms for development of non-invasive visual biomarkers of aging

**DOI:** 10.18632/aging.101629

**Published:** 2018-11-09

**Authors:** Eugene Bobrov, Anastasia Georgievskaya, Konstantin Kiselev, Artem Sevastopolsky, Alex Zhavoronkov, Sergey Gurov, Konstantin Rudakov, Maria del Pilar Bonilla Tobar, Sören Jaspers, Sven Clemann

**Affiliations:** 1 HautAI OU, Tallinn, Estonia; 2 Lomonosov Moscow State University, Moscow, Russia; 3 Federal Research Center “Computer Science and Control” of the Russian Academy of Sciences, Moscow, Russia; 4 Skolkovo Institute of Science and Technology, Moscow, Russia; 5 Insilico Medicine, Rockville, MD 20850, USA; 6 The Buck Institute for Research on Aging, Novato, CA 94945, USA; 7 The Biogerontology Research Foundation, London, UK; 8 Beiersdorf AG, Hamburg, Germany; * Equal contribution

**Keywords:** photographic aging clock, photographic aging biomarker, age prediction, biomedical imaging, computer vision, deep learning

## Abstract

Aging biomarkers are the qualitative and quantitative indicators of the aging processes of the human body. Estimation of biological age is important for assessing the physiological state of an organism. The advent of machine learning lead to the development of the many age predictors commonly referred to as the “aging clocks” varying in biological relevance, ease of use, cost, actionability, interpretability, and applications. Here we present and investigate a novel non-invasive class of visual photographic biomarkers of aging. We developed a simple and accurate predictor of chronological age using just the anonymized images of eye corners called the PhotoAgeClock. Deep neural networks were trained on 8414 anonymized high-resolution images of eye corners labeled with the correct chronological age. For people within the age range of 20 to 80 in a specific population, the model was able to achieve a mean absolute error of 2.3 years and 95% Pearson and Spearman correlation.

## Introduction

One of the critical challenges in aging and longevity research and healthcare in general is the development of widely-available and reliable biomarkers of aging. Individuals can be of the same chronological age and have different biological ages. Biological age reflects functional capability and physiological status, whereas perceived age reflects the age other people guess a person to be [1]. Chronological age predictors can be used to identify the divergence between estimated biological or perceived age and true chronological age among people with accelerated or delayed aging [1]. It is important to develop non-invasive photographic biomarkers, since they are capable of providing valuable insights about the condition of the human body. Highly-accurate predictors of chronological age can also be used to evaluate the various lifestyle, medical, and cosmetic interventions.

Humans can predict the age of other humans with reasonable accuracy. However, people’s error rates vary across ethnic groups and from person to person. The many diseases as well as the general health status of the person are often apparent to the trained professionals, family members and untrained individuals. Humans without prior medical training can detect a variety of acute diseases using just facial images [2].

In order to investigate the biological relevance of photographic biomarkers of biological age, the accurate chronological age predictors must be developed and studied. In this study, the photographic images of human skin were used to predict age. Wrinkles and changes in skin pigmentation indicate aging making skin condition a reasonably good predictor of chronological age (here and below the age refers to the chronological age). Our main finding was that the photographic images of the skin around the eye can serve as a very accurate, non-invasive biomarker of the age. We also found that this photographic aging clock was able to estimate age with higher precision than the methylation aging clock commonly referred to as the Horvath’s clock [3]. Horvath’s clock is a DNA-based epigenetic measure which is considered to be a state-of-the-art aging biomarker. While age predictors such as the methylation aging clock are accurate in predicting the chronological age and multiple studies suggest the biological relevance of these clocks, there are some questions regarding the biological utility of and the relationship between these predictors [4].

There are numerous papers on age prediction with biomarkers; these papers cover a broad range of disciplines including biology, bioinformatics, machine learning and computer vision. The use of the facial images for age estimation is widespread. This approach is supported by a large number of images of faces and datasets of faces available on the internet, such as FG-NET [5], MORPH (the largest public face aging dataset) [6], Adience [7], and IMDB-Wiki [8]. The predictors trained on imaging data are commonly used for related tasks, such as: gender estimation, landmark estimation, and 3D model reconstruction. Most computation methods invented in the last decade rely on statistical models and manually-designed features [9–12], which are generally useful only for specific tasks [13]. However, with advances in computer vision, age estimation can even be done on unconstrained (in-the-wild real-life) images. Unconstrained images are images that may contain artifacts such as blur, occlusion, or various degrees of deformation. Deformation can occur from either strong facial expressions or head movement. Qawaqneh et al. [14] employed the VGG-Face network for age prediction. Their solution achieves high accuracy of age prediction on Adience in-the-wild dataset (59.9 exact accuracy and 90.57 1-off accuracy for 8 age groups). Zhang et al. [15] proposed a solution for simultaneous age and gender prediction with Residual Networks of Residual Networks (RoR) pre-trained on the ImageNet [16] and the IMDB-WIKI [8] datasets. Their solution achieves very high quality on the Adience dataset (66.7 exact accuracy and 97.38 1-off accuracy) with a 34-layer network. Rothe et al. [8] studied the tasks of chronological (real) and apparent (perceived by other people) age prediction. Their pipeline started with a face detector which determined the position of a face on an input image, the position was then normalized (without using facial landmarks). Next, classification occured *via* the convolution neural network (CNN) pre-trained on ImageNet dataset and fine-tuned on the IMDB-Wiki dataset. The authors reported the MAE of 3.318 years for apparent age prediction on IMDB-Wiki dataset and MAE 2.68 and 3.09 years for chronological age prediction on MORPH2, and FG-NET datasets, respectively. Another application of using face images as a biomarker of aging involves imposing age changes on the image using Deep Feature Interpolation [17] and Generative Adversarial Networks (GANs) [18]. It is possible to produce an older image of a person from a recent photograph with high quality results [17,18]. One of the popular approaches to age estimation is based on the analysis of various biological data types. For instance, Zhavoronkov et al. [19] predicted age by training the ensembles of deep neural networks (DNN) on the basic blood biochemistry data. These models were trained on over 60,000 blood biochemistry tests samples. The authors reported the values R^2^ = 0.82 and MAE = 5.55 years for chronological age prediction. The ensemble of DNNs also identified the five most important markers for predicting human chronological age: albumin, glucose and urea concentration, alkaline phosphatase activity, and erythrocytes number. Further studies of this approach helped establish its biological relevance by testing the modified predictor on the large population data sets including the data from the National Health and Nutrition Examination Survey (NHANES) and demonstrate that the DNN-based age-predictors can be population-specific by comparing the accuracy of the predictor in the Canadian, Korean and Eastern European Populations [20]. Age can also be predicted by DNA methylation rate, as was discussed in this seminal paper [21]. In this paper, using of 8,000 samples from 82 Illumina DNA methylation array datasets, encompassing 51 healthy tissues and cell types, allowed age to be predicted with R^2^ = 0.96 (between methylation-base predicted age and true chronological age) and MAE = 2.7 years. Despite high precision, the main disadvantage of DNA methylation-based methods is their invasive nature [5,21].

Our work is devoted to usage of deep learning approach for accurate chronological age prediction and investigation of features contributing to age prediction. This method only requires a single high-resolution photo of the corner eye area. The eye corner area of the human face is believed to be the most prone to aging [22]. Therefore, we believe it holds important clues for the creation of photographic aging biomarkers.

## RESULTS

### Neural networks training

The best Xception-based model achieved a MAE of 2.38 and of 2.30 years before and after skip-connection, respectively (see Table 1). Table 2 contains the accuracy evaluations of the predictions for the various age groups on Adience dataset.

**Table 1:** Comparison of the best described approaches of age estimation and their accuracy assumed by MAE, years.

***Approach name***	***Dataset***	***MAE, years***
Xception (this work)	Eye corners photos	2.38
Xception with skip-connections (this work)	Eye corners photos	2.30
VGG [8]	FG-NET	3.09
VGG [8]	MORPH	2.68
SVR on Gabor filters [5]	FG-NET	3.17
Penalized regression model [21]	DNA-methylation data	2.70
Ensemble of 21 DNN [19]	Blood sample test	5.55

**Table 2:** Exact and 1-off accuracy of age estimation (this work) for Adience dataset age groups.

Age group (years range)	Exact accuracy	1-off accuracy
25-32	0.68	0.98
33-38	0.50	1.00
38-44	0.63	0.95
44-48	0.55	0.92
48-54	0.60	0.97
54-60	0.54	0.99
60-69	0.78	0.98

1-off accuracy represents the accuracy when the result is off by 1 adjacent age label left or right [7].

**Figure 1:**
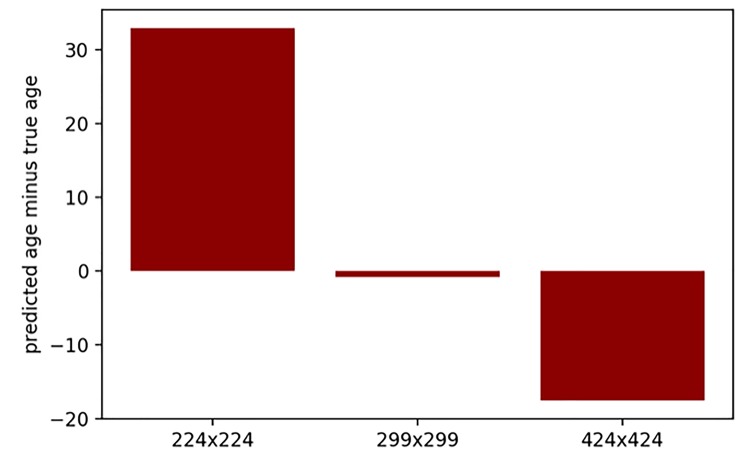
**Prediction error (predicted age minus true age) for the same 25 images with various resolutions.** Images were passed through the developed neural network, with kernels trained for 299 x 299 pixels resolution.

The neural network model for age prediction was designed to accept images of an arbitrary resolution, then the convolutional layers applied kernels of fixed size to the image regions. Lower resolution images had relatively sharper color transitions, for the convolutional kernels this corresponded to rougher skin look. On the other hand, higher image resolution corresponded to smoother colors and better smooth-looking skin. Age was greatly overestimated for the lower-resolution images (224 x 224 pixels) and greatly underestimated for the higher-resolution images (424 x 424 pixels) when passed through the developed neural network, with kernels trained for 299 x 299 pixels resolution (see Fig. 1). Therefore, it seems that the model heavily depends on skin conditions, such as wrinkles and pigmentation. For downsampled images, convolutional kernels coincided with larger areas of the image, which corresponded to relatively more smooth skin. For upsampled images this pattern was reversed.

### Effect of area occlusion on the prediction quality

**Figure 2:**
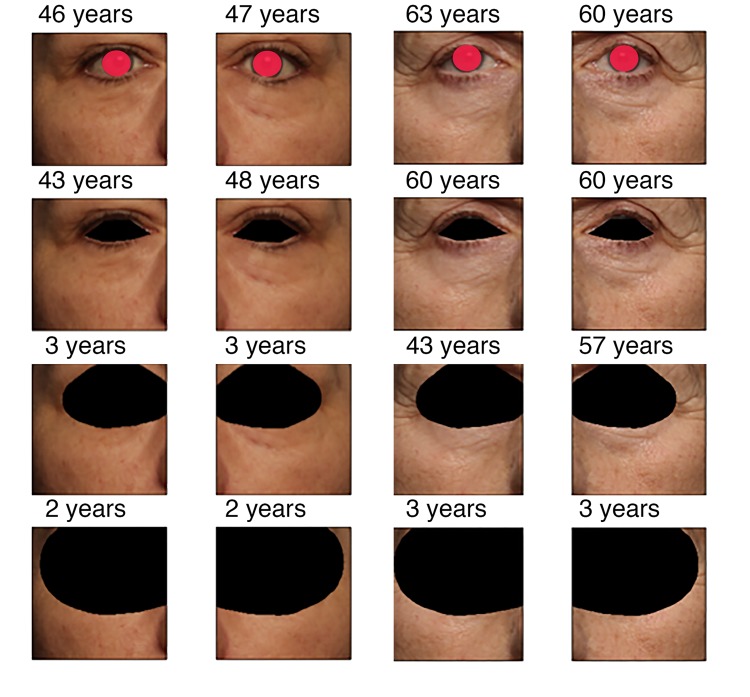
**Predicted age vs. the extent of occlusion for two persons.** Picture order (up to bottom): original, covered eye area, eyelid and corner covered, and half image area covered. See text for clarifications. Real chronological age for the left subject is 50 years, for the right subject is 62 years.

**Figure 3:**
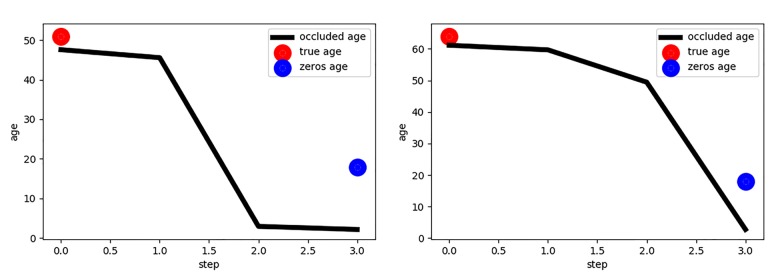
**Estimated age vs. the occlusion step for two persons.** The first plot represents the results for the younger-aged person (50 years). The second plot represents the results for the older-aged person (62 years). Blue points correspond to the age produced by zeros tensor. This age reflects the initial step of age estimation by neural network model when it was fed an all-black image. This happened because of learned biases parameters.

**Figure 4:**
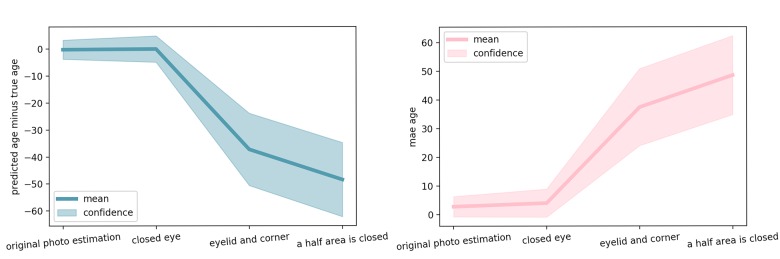
**Estimation error for several significant steps of occlusion.** Mean and standard deviation of the error over 165 pairs of validation images (left and right eye) is reported.

The progression of the prediction error with the image area occluded is shown on Fig. 2 for two people of different age. When only the eye area is closed, the accuracy of age prediction is almost same as for the original not occluded image. For the picture of the subject of younger age (50 years) the accuracy decreases dramatically for the image with occluded eyelid area and eye corner area, the decrease in accuracy is less significant for the older adult (62 years). When half of the eye corner image is occluded, the accuracy falls dramatically for older age subjects as well. These results are represented in Fig. 3 as a qualitative evaluation. Same trend was observed on a number of subjects (see Fig. 4).

### Validation of the algorithm

**Figure 5:**
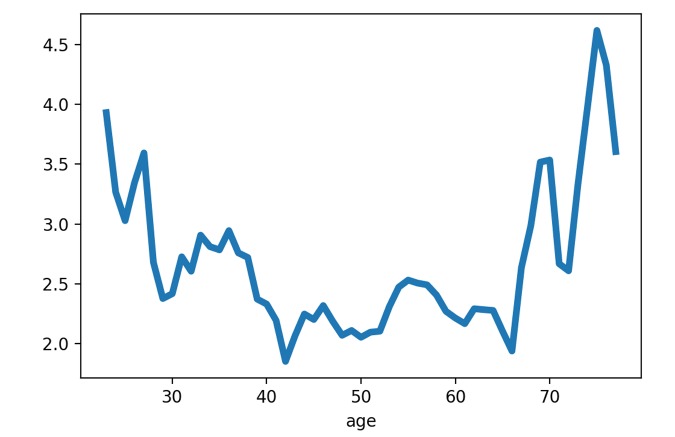
PhotoAgeClock predicted age error for the test set within different ages.

Fig. 5 contains the distribution of prediction error (MAE) with regard to the (w.r.t) age group. The distribution was calculated empirically by moving average window on four points of age bins. The plot shows that prediction error is the lowest for the age range of 40-65; for the age range of 20-40 and 65+ years age prediction error was relatively higher.

## DISCUSSION

### Comparison with other age prediction approaches

The data for the accuracy of age prediction by the best known methods is compared in Table 1. The results clearly indicate that high-resolution information about eye corner wrinkles can be utilized for accurate chronological age estimation. We believe our approach is beneficial for age prediction due to its non-invasive nature, ability to work with anonymized data and high accuracy.

### Investigation of features contributing to age prediction

The following procedures were performed in order to detect the areas most sensitive for the age changes. In the experiment, the eyes were occluded with black markings of various sizes. The eye corner and eyelid areas influenced the predicted age the most. When occluded, the absence of these areas produced the highest relative error. The experiment implicates that the wrinkles in the eye region contained the most important features for age prediction suggesting that these areas may be used for development of candidate photographic aging biomarkers. It is very important to emphasize that the omitted area of the eye affected the age prediction only to a small extent. Fig. 2 and Fig. 3 demonstrate that for the middle-age person (50 years old), right after the third occlusion step which corresponds to eyelid and eye corner covered, predicted age falls to the value as when neural network model was fed an all black image (“zeros age”). For the older person (62 years old) the predicted age falls slower w.r.t. image area occluded. The same effect was observed for many different images (see Fig. 4). We believe this happens since age-related wrinkles were more evenly distributed across the face skin of the elderly person. Another candidate photographic biomarker is skin pigmentation of the malar area.

### Accuracy prediction for various age groups

The age prediction error was quite uniform for persons within age group 40-65 years. It was observed that age prediction error was higher for ages range 20-40 years and range 65+. The most possible reason is that these age groups were represented to a lesser extent in the dataset. Another possible reason for larger error in senior age group is that divergence in human phenotypes becomes larger as they age. People of the same senior age can look much younger or older than they actually are as they age at a different rate. Aging rate, presence of wrinkles and pigmentation, which we believe are photographic biomarkers of aging depends a lot on the lifestyle.

**Figure 6:**
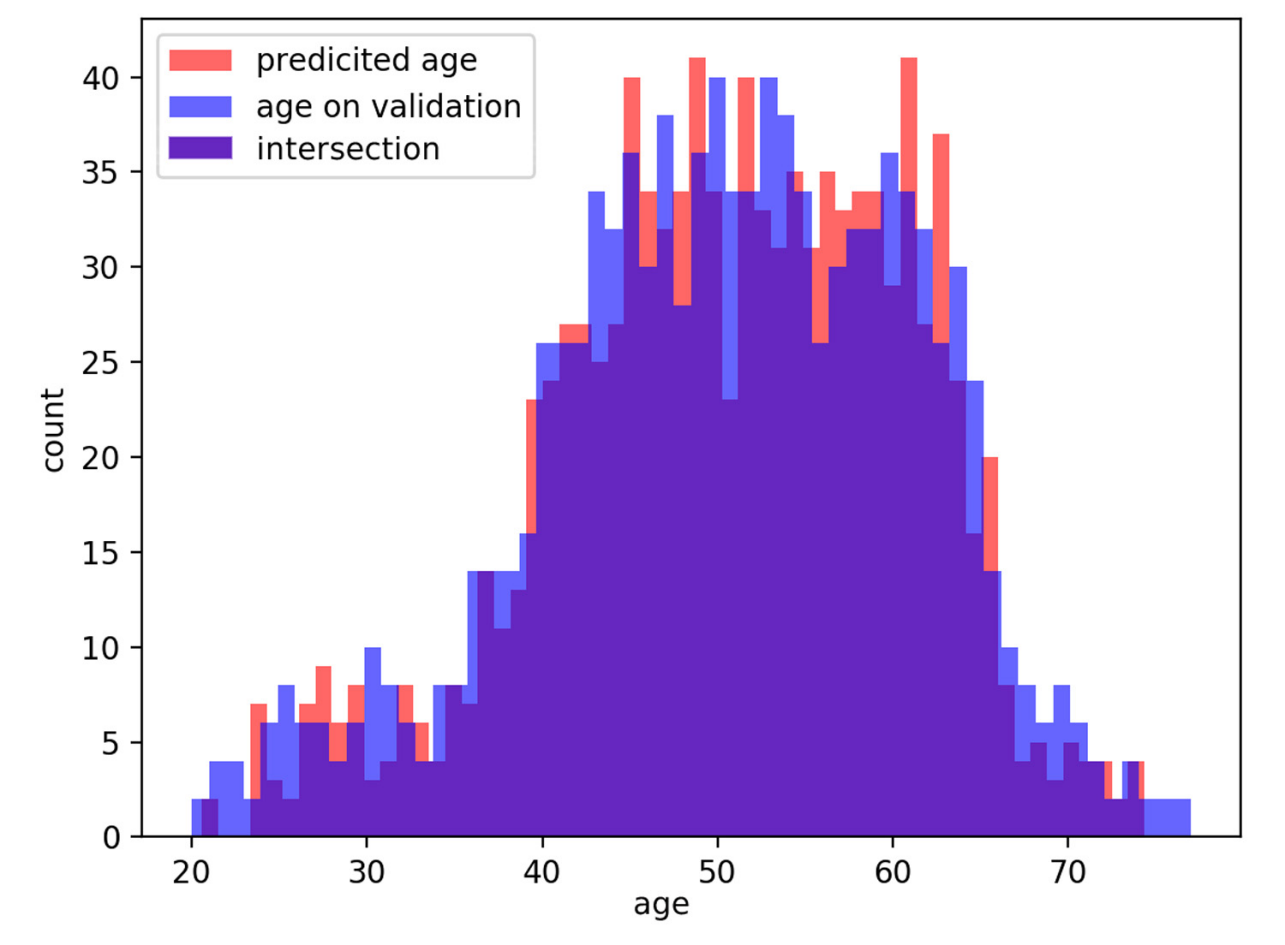
Distribution of actual age in the dataset and predicted age (PhotoAgeClock) labels in the validation set.

The distributions of predicted age labels in the validation dataset resembles the age distribution in the dataset (see Fig. 6).

### Algorithm validation

We considered several measures of predictive accuracy: mean average error (MAE), Pearson correlation coefficient and Spearman correlation coefficient. MAE refers to the results of measuring the difference between PhotoAgeClock predictions and chronological age. We got MAE of 2.3 years on validation set, which indicates that average sum of all absolute errors between PhotoAgeClock and chronological age on all instances of the validation dataset is 2.3 years.

**Figure 7:**
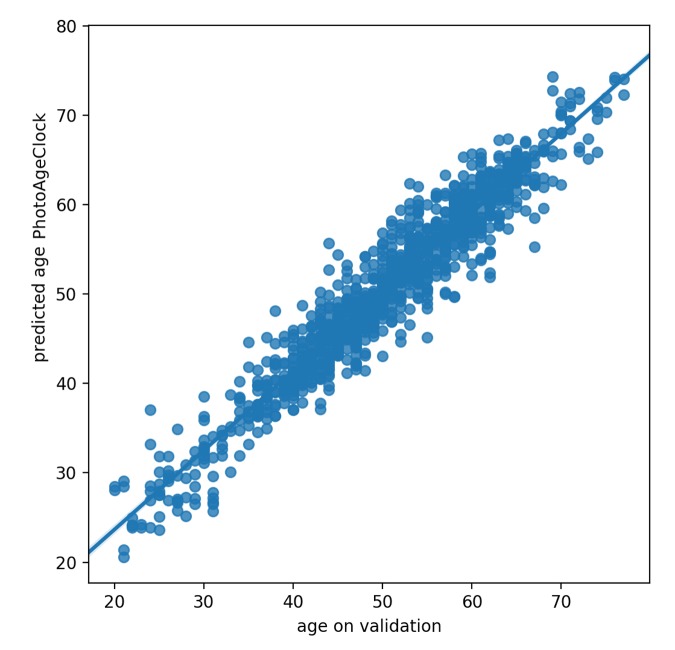
Correlation between predicted age and actual age on validation dataset.

Correlation coefficients describe the strength and the direction of the relationship of PhotoAgeClock and chronological age, whereas Pearson correlation coefficient evaluates linear relations and Spearman correlation coefficient evaluates monotonic relations. Pearson correlation coefficient between the actual and predicted values by PhotoAgeClock on validation dataset was equal to 0.96 and Spearman correlation coefficient was equal to 0.95 (see Fig. 7). According to these accuracy measures, PhotoAgeClock performs well on high-resolution dataset of eye pictures.

### Other applications

**Figure 8:**
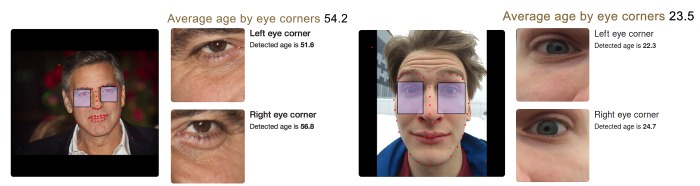
**Algorithm performance on images obtained with professional cameras and mobile devices.** (left) Algorithm performance on a high resolution photo of a celebrity (George Clooney). Chronological age of the person for the time when the picture was taken was 53 years, predicted age by two eye corner areas is 54.2 years. Editorial credit: Denis Makarenko / Shutterstock.com. (right) Algorithm performance on photo obtained with frontal camera of mobile device (selfie). Chronological age of the person is 22, predicted age by two eye corner areas is 23.5. The skin of eye area is smooth enough and young age is recognized despite the strong face expression.

A special web demo was created to conduct a quality assessment of the constructed PhotoAgeClock. PhotoAgeClock showed a significant extent of domain invariance and can be applied to arbitrary high-resolution photos that contain a full face of person (see Fig. 8 left) and even photos obtained with frontal cameras of mobile devices (see Fig.8 right).

In Fig. 8 (left) the right eye produced a relatively higher age of 56,8 years. The left eye produced a relatively lower age of 51,6 years. The reason for this discrepancy may be the difference in the number of wrinkles around the eyes in this photo.

Fig. 8 (right) demonstrates PhotoAgeClock can accurately recognized age even despite the strong face expression.

### Limitations

We represent a novel non-invasive biomarker of aging and demonstrate that PhotoAgeClock can predict chronological age with quite high accuracy on the specific dataset of high-resolution eye corner images. However, the true biomarker of aging needs to show the association with morbidity or mortality, and this analysis requires experiments on retrospective data.

PhotoAgeClock demonstrates the potential to be utilized for assessment of high-quality pictures obtained with devices like professional cameras and smartphones (see Fig. 8), nevertheless, PhotoAgeClock works best on standardized high-resolution images of eye corners.

## MATERIALS AND METHODS

### The dataset

The dataset consisted of 8,414 anonymized high-resolution left and right eye corner photos of caucasian females with labeled true chronological age. The dataset was split into a training set and a test set at the proportion of 7:1. Training set and the test set contained only images for different people to avoid overfitting. Images of the left and the right eye corners of the same person were put together, either in the training set or in the test set. The age of the individs was in the range of 20-80 years. Initially, images size was 2258 x 1506 pixels. The age distribution in the dataset used was uneven: the age range of 40-65 years was represented to a higher extent (see Fig. 6). During training, we sampled images at probabilities equal to the inverse frequencies of their ages. Thus, the images of persons with each age were presented to the neural network with the same frequency. It made the model suited to work with all age groups from the dataset.

### Image pre-processing

The input images were resized to 299 x 299 pixels, to fit the DNN input dimension, and color intensities were linearly translated to a range [-1; 1]. They contained facial skin details such as wrinkles and pigmentation which are important features of aging process.

In addition, the images from test dataset were resized from their original resolutions: to 424 x 424 pixels, to the standard resolution for our neural network (299 x 299 pixels), and to a lower resolution (224 x 224 pixels — commonly used as a standard for ResNet [23]).

The following data augmentation methods were applied to increase the dataset size: horizontal and vertical mirroring, rotation up to ±10◦, horizontal and vertical shifts up to ±15% of width and height, respectively, zoom from 70% to 130% of image size, and affine shear with an angle up to ±0.5 radians. The quantitative degree of each elementary image transformation was picked uniformly from the aforementioned parameter ranges.

### Algorithm development

Our approach aimed to solve the task of age prediction. We used Xception [24], a DNN-based model. In this neural network model all layers, except the last fully-connected (dense) layer, were initialized with pre-trained weights from the ImageNet [16] dataset. We modified the model to increase its quality in the following manner. We added skip-connections from each residual block to the dense output layer and changed the last layer to fit the regression model. We used the ADAM optimization algorithm [25] and MSE loss function for training. The best quality was reached after 150 epochs.

**Figure 9:**
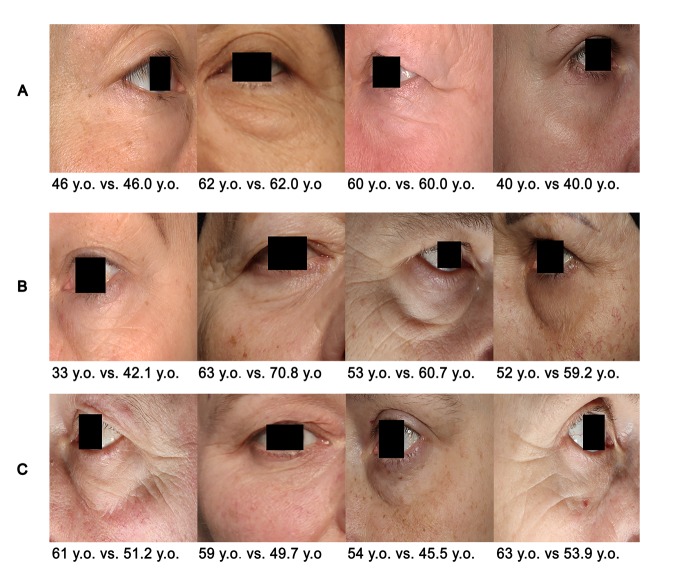
**Examples of PhotoAgeClock performance.** (**A**) Cases when the trained model produced the lowest errors on the test set. (**B**) Cases when the trained model overestimated age the most on the test set. (**C**) Cases when the trained model underestimated the age the most on the test set. True vs. predicted age is labeled. Eye areas were erased for anonymity purposes but were present in the actual dataset pictures.

Figs. 9 A-C visually represent the cases of the algorithm application when PhotoAgeClock: 1. predicts age correctly (Fig. 9A), 2. overestimates age the most (Fig. 9B) and 3. underestimates age the most (Fig. 9C).

### Algorithm validation

We used MAE to evaluate algorithm accuracy. Pearson correlation coefficient and Spearman correlation coefficient were used to measure correlations between actual age and predicted age by PhotoAgeClock.

### Screening of the images for the pattern more sensitive for age changes

In order to evaluate how the predicted age estimation changed when a certain fraction of the area of the image was occluded, we detected eye landmarks with Dlib library [26]. Then the color of pixels around the eye border was changed to black. The difference between the age predicted for the images without the black pixels and images occluded area with black pixels was calculated. Photos of the left and right eyes for two different age people with different occlusion steps are presented in Fig. 2. For this paper the eye areas were covered with red markings for anonymity purposes. However, in the he actual dataset the eye areas were not covered.

## CONCLUSION

In this study we developed a deep-learning network that uses photographs of eye corners from facial images to predict human chronological age referred to as the PhotoAgeClock. We demonstrated that by making use of the current advances in deep learning and computer vision, it is possible to achieve very high quality age estimations based only on the information from a small facial region (the eye and the skin area around the eye). These findings have several important implications. Firstly, when estimating age from facial images, high-resolution images are very beneficial. Secondly, wrinkles and skin pigmentation serve as reliable non-invasive visual biomarkers of aging, thus, they can be used as a source of valuable insight into the condition of the human body and health. Thirdly, as accuracy of age prediction does not decrease significantly when occluding the eye area PhotoAgeClock can be utilized to predict age on anonymized datasets of images.

We also found that, compared to other regions on the investigated images, the trained model considers the skin around the eye to be the most age-relevant area. Based on these findings, we believe that it is prudent for future studies to explore what information pattern recognition based on the condition of human skin can provide.

We hypothesized that deep learning systems trained on large numbers of annotated human facial images could outperform humans in predicting various diseases and aging. While aging by itself is not likely to be classified as a disease [27], many human diseases are closely correlated with age. It may be possible to use these biomarkers of aging to provide early detection and prevent the onset of a variety of diseases. The emergence of the many credible geroprotectors [28], including senolytics [29], NAD-pathway modulators [30–32], metformin [33], rapamycin and other TORC inhibitors [34,35], their natural mimetics [36] and the many techniques for identifying novel interventions [37] calls for the rapid assessment of efficacy and safety and any panel of aging biomarkers can be easily augmented with the PhotoAgeClock and other non-invasive photographic predictors of age. We believe the main value of PhotoAgeClock and other imaging biomarkers trained on skin imaging data is in estimation of the differential changes induced by the various interventions including cosmetic, lifestyle, and medical and establishing the correlations between the many other aging clocks that are rapidly emerging. The photographic aging clocks may also help with the development of biomedical interventions and skin care treatments for the individual health status, skin type, climate, geography and other parameters and personalize the treatments for each individual.
